# Higher numbers of circulating endothelial progenitor cells in stroke patients with intracranial arterial stenosis

**DOI:** 10.1186/1471-2377-13-161

**Published:** 2013-11-05

**Authors:** Zhizhong Liu, Xiurong Ding, Fang Fang, Ruimin Wang, Yan Chen, Yuetao Ma, Guojun Zhang, Xixiong Kang

**Affiliations:** 1 The Centre for Laboratory Diagnosis, Beijing Tiantan Hospital, Capital Medical University, Beijing, 100050, China; 2 Clinical Laboratory Centre, Beijing Friendship Hospital, Capital Medical University, Beijing, 100050, China; 3 Department of Neurology, Beijing Tiantan Hospital, Capital Medical University, Beijing, 100050, China

**Keywords:** Intracranial artery stenosis, Atherosclerosis, Circulating endothelial progenitor cells, Fibrinogen

## Abstract

**Background:**

Bone marrow-derived endothelial stem cells participate in vascular repairs. Numbers of circulating endothelial progenitor cells (cEPCs) are associated with atherosclerosis. Fibrinogen plays a key role in atherosclerosis. Objective was to assess if cEPC counts were associated with atherosclerotic intracranial artery stenosis (IAS).

**Methods:**

Three hundred subjects (108 patients with stroke and IAS (IAS), 120 control patients with stroke without IAS (CP), and 72 healthy controls (HC)) were retrospectively analyzed. cEPCs were identified and counted by flow cytometry using CD34, CD133 and KDR. Plasma fibrinogen was measured by immunoturbidimetry. cEPC counts were compared between the three groups.

**Results:**

cEPC numbers were significantly higher in IAS (0.059 ± 0.031%) than in CP (0.026 ± 0.012%) (*P* < 0.001) and HC (0.021 ± 0.011%) (*P* < 0.001), but without difference between CP and HC (*P* = 0.401). Multiple logistic regression analysis showed that cEPC levels (OR 3.31, 95%CI 1.26-8.87, *P* = 0.025; IAS *vs.* CP) were independent markers of IAS after adjustment for hypertension, diabetes and smoking. No significant correlation between cEPC counts and plasma fibrinogen levels was observed (*P >* 0.05).

**Conclusion:**

cEPC numbers were associated with degrees of IAS. This measurement may be useful for non-invasive evaluation of atherosclerotic IAS.

## Background

Intracranial arterial stenosis (IAS) is an important cause of stroke. The number of intracranial symptomatic vessels and their stenosis degree is associated with a higher risk of stroke recurrence and other major ischemic events [1-4]. However, the mechanisms for atherosclerosis initiation and progression in intracranial large arteries are still ill understood. First, intracranial atherosclerosis may be characterized by multiple stenoses of cerebral large arteries, which may result in chronic brain hypoperfusion, responsible for an enhanced angiogenic response [5]. Secondly, endothelial repair may be crucial for atherosclerosis progression in intracranial large arteries [6]. In addition, bone marrow-derived endothelial stem cells can differentiate into mature vascular endothelial cells and participate in vascular repair [7].

Many factors, including proinflammatory interleukin (IL)-8 [8] and chemokine monocyte chemoattractant protein-1 (MCP-1) [9], are important for endothelial progenitor cells (EPCs) mobilization. Furthermore, even if their exact role is not yet completely understood, circulating EPCs (cEPCs) are involved in vascular pathologies [10-13]. Elevated cEPC levels have been associated with atherosclerotic diseases, but the results are conflicting [14-20]. Serum IL-8 and MCP-1 levels are elevated in pathological conditions, along with elevated numbers of cEPCs [12,18,21-23]. Fibrinogen exposure can result in up-regulation of expression and secretion of MCP-1 and IL-8 in endothelial [24] and dendritic cells [25]. Fibrinogen plays a key role in the development of atherosclerosis [26].

We hypothesized that there might be a potential association between plasma fibrinogen levels, cEPCs and IAS. This present study explored the association between cEPC numbers and IAS, and if there was a correlation between EPC and fibrinogen levels. cEPCs might be a disease marker in IAS, and may also be a treatment target in IAS.

## Methods

### Patient demographics and exclusion criteria

This study was approved by the Ethics Committee of the Beijing Tiantan Hospital Affiliated to the Capital Medical University, China (approval #KYLW2013-004-01), and individual informed consent was waived. A cohort of 108 patients with symptomatic IAS, 120 control patients without IAS (CP) and 72 healthy controls (HC) from September 2011 to July 2012 were retrospectively analyzed. IAS and CP patients were selected from patients with a first-ever ischemic stroke in the neurology department of the Beijng Tiantan Hospital. Intra- and extracranial cerebral large arteries were evaluated by trans-cranial Doppler (TCD) in all subjects. IAS was reconfirmed by digital subtraction angiography (DSA). Exclusion criteria were: 1) modified Rankin Scale score > 2; 2) emboligenic cardiopathy; 3) cancer diagnosis; 4) chronic inflammatory disease; or 5) non-atherosclerotic intracranial stenosis, such as Sneddon syndrome, Moya-Moya disease, postradiotherapy angiopathy, and vasculitis.

### Image acquisition

TCD was performed using a MultiDop-X/TCD (Compumedics DWL, Singen, Germany), with a hand-held transducer in a range-gated, pulsed-wave mode at a frequency of 2 Mhz. IAS was diagnosed according to validated criteria [27]. Cervical intracranial artery (ICA) atherosclerosis was regarded as any ICAs showing a stenosis ≥50%.

Intracranial stenosis was reconfirmed by DSA (Innova 3100IQ, GE Healthcare, Waukesha, WI, USA,) and was defined as a focal stenosis ≥50% in luminal reduction affecting the main cerebral large arteries. Stenosis was classified as severe (≥70%) or non-severe (<70%), based on the symptomatic narrowed artery [1].

### Flow cytometry

Peripheral blood (2 mL) was collected in tubes containing K_2_-EDTA on the 7^th^ day after acute onset and was processed within 1 hour of collection. Peripheral blood mononuclear cells (PBMCs) were isolated by density gradient centrifugation using Ficoll-Hypaque (Amersham, GE Healthcare, Waukesha, WI, USA), washed with PBS, and counted for recovery and viability using Trypan Blue. Since EPCs are characterized by the co-expression of CD34, CD133 and KDR [28,29], we determined the proportion of cEPCs in PBMCs by flow cytometry using a triple staining with fluorescein-conjugated monoclonal antibodies against these markers. Briefly, PBMCs (1 × 10^6^) were incubated with CD34-PerCP-Cy5.5 (Becton Dickinson, Franklin Lake, NJ, USA), CD133-PE (eBioscience, San Diego, CA, USA), and KDR-Alexa Flour647 (Becton Dickinson, USA) for 25 min at 4°C in a dark room. Non-specific binding was determined by staining an aliquot of cells with fluorescein-conjugated IgG1 and IgG2a isotype controls (Becton Dickinson, Franklin Lake, NJ, USA). After washing once with PBS, cells were fixed with 1% paraformaldehyde and analyzed using a FACS Calibur flow cytometer (Becton Dickinson, USA). We gated CD34+ peripheral blood cells in the mononuclear cell fraction, followed by the examination of the resulting subpopulation for expression of KDR, CD133 or both. The frequency of peripheral blood cells positive for these markers was determined by a 2-dimensional side-scatter fluorescence dot-plot analysis, after appropriate gating. A minimum of 50,000 events was acquired for each sample. Data were analyzed using the embedded Cellquest software. EPC numbers were expressed as the proportion of total PBMCs.

### Plasma fibrinogen levels

On the 7^th^ day after acute onset, peripheral blood (3 mL) was collected in tubes containing sodium citrate to measure plasma fibrinogen levels, using a CA5000 Coagulation Analyzer (Sysmex, Kobe, Japan).

### Statistical analysis

Inter-groups differences were assessed using the χ^2^, Fisher’s exact tests, Student’s *t*-test or one-way ANOVA with post hoc analysis. Pearson correlation analysis was used to analyze the relationship between plasma fibrinogen levels and cEPC numbers. A multiple logistic regression model was used to identify independent IAS markers (variables with a *P*-value <0.05 in univariate testing were included); adjustment for age, gender, and vascular risk factors was also performed. Results are expressed as adjusted odds ratios (OR) and corresponding 95% confidence intervals (CI). *P* < 0.05 was considered to be statistically significant. All analyses were performed using SPSS 13.0 (SPSS Inc., Chicago, IL, USA).

## Results

### Patients’ characteristics

Table 1 presents subjects’ demographics and risk factor characteristics. The proportion of patients with hypertension, diabetes and tobacco use were higher in the IAS group (all *P* < 0.001). A total of 206 stenoses in 108 IAS patients were identified.

**Table 1:** Patients’ demographic and risk factor characteristics

	**HC (n = 72)**	**CP (n = 120)**	**IAS (n = 108)**	**P**^**Δ**^	**P**^**#**^	P^*^
Age, mean ± SD	56.0 ± 10.2	56.9 ± 7.6	61.5 ± 12.1	0.652	0.132	0.281
Gender (female), n (%)	28 (38.9)	48 (40.0)	48 (44.4)	0.879	0.460	0.497
BMI, Kg/m2	24.2 ± 4.2	26.1 ± 8.6	26.9 ± 8.9	0.035	0.031	0.756
sBP, mmHg	123.2 ± 17.7	134.1 ± 16.54	138.1 ± 18.72	0.065	0.036	0.452
dBP, mmHg	81.6 ± 10.1	83.52 ± 12.05	85.6 ± 10.1	0.396	0.041	0.462
TG, mmol/l	1.325 ± 0.35	1.446 ± 0.77	1.504 ± 0.83	0.689	0.478	0.837
TC, mmol/l	3.935 ± 0.93	4.078 ± 0.68	4.697 ± 0.85	0.696	0.019	0.089
HDL, mmol/l	1.198 ± 0.26	1.05 ± 0.16	0.955 ± 0.15	0.099	0.003	0.297
LDL, mmol/l	2.43 ± 0.81	2.626 ± 0.55	2.624 ± 0.78	0.623	0.601	0.991
Glu, mmol/l	5.75 ± 1.06	6.059 ± 3.32	5.58 ± 0.97	0.745	0.748	0.555
CRP, mg/l	0.65 (0.31-1.37)	1.55 (0.65-2.71)	2.75(1.2-3.25)	0.021	<0.001	0.012
Hypertension, n (%)	0	30 (25.0)	84 (77.8)	-	-	<0.001
cEPCs (%)						
CD34+	0.293 ± 0.182	0.364 ± 0.201	0.553 ± 0.311	0.028	0.009	0.019
CD34+/CD133+	0.098 ± 0.032	0.113 ± 0.064	0.246 ± 0.142	0.382	<0.001	<0.001
CD34+/CD133+/KDR+	0.021 ± 0.011	0.026 ± 0.012	0.059 ± 0.031	0.401	<0.001	<0.001
Diabetes, n (%)	0	6 (5.0)	24 (22.2)	-	-	<0.001
Hyperlipidemia, n (%)	0	18 (15.0)	24 (22.2)	-	-	0.160
CAD, n (%)	0	12 (10.0)	18 (16.7)	-	-	0.137
Smoking, n (%)	0	48 (40.0)	78 (72.2)	-	-	<0.001
Drug treatment				-	-	
Statin, n (%)	4 (5)	31 (26)	35 (32)	-	-	0.316
ACE inhibitor, n (%)	-	20 (17)	31 (29)	-	-	0.029
Angiotensin II receptor blocker, n (%)	-	3 (2)	2 (2)	-	-	0.739
Beta blocker, n (%)	-	10 (8)	13 (12)	-	-	0.345
Calcium antagonist, n (%)	-	19 (16)	34 (32)	-	-	0.005

P^Δ^: CP vs. HC P^#^: IAS vs. HC P^*^: IAS vs. CP.

sBP: systolic blood pressure; dBP: diastolic blood pressure.

HC, healthy controls; CP, control patients; IAS, patients with intracranial artery stenosis; CAD: coronary artery disease.

### Endothelial progenitor cells

**Figure 1:**
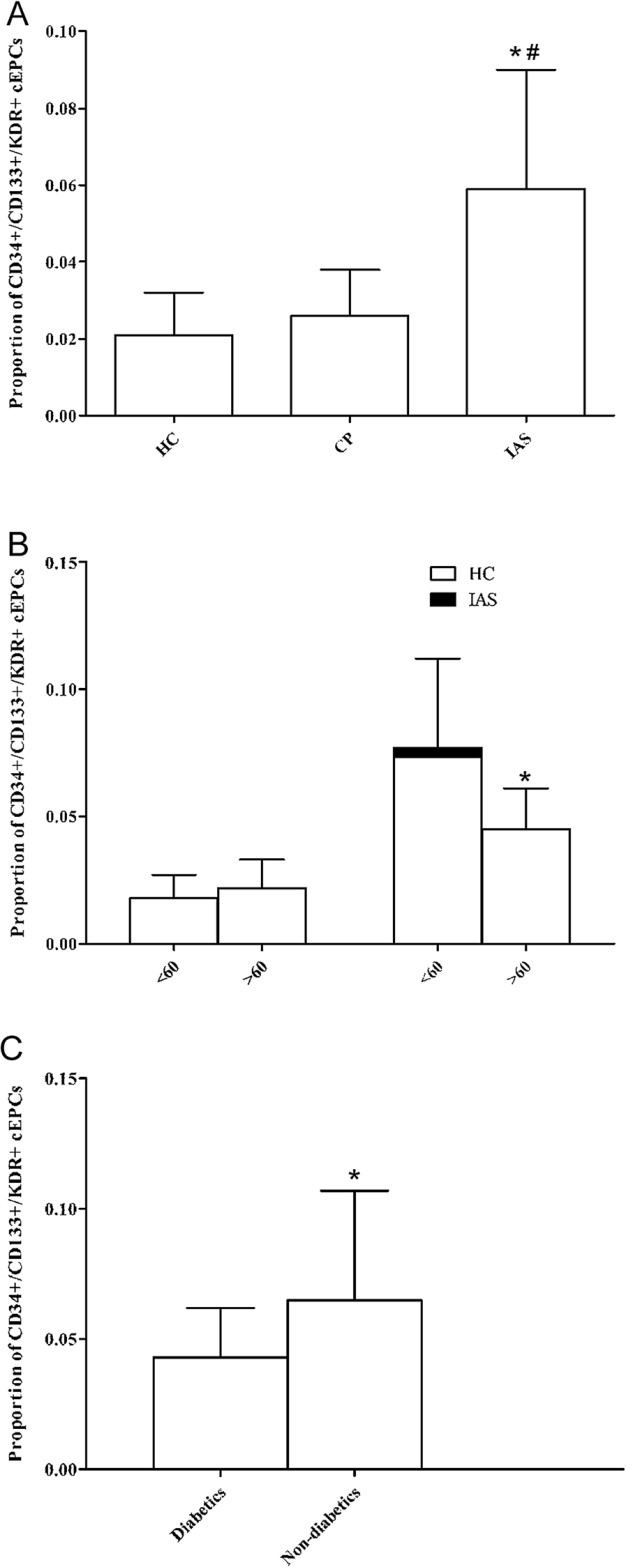
**cEPCs proportions across different groups. (A)** cEPC levels in the peripheral blood of HC, CP and IAS (*P < 0.01, IAS *vs.* HC; # *P* < 0.01, IAS *vs.* CP). Rresults are presented as mean ± SD. **(B)** Differences in cEPC levels in the peripheral blood of HC and IAS according to age (*P < 0.05, <60 *vs.* ≥60 years old in IAS patients). Results are presented as mean ± SD. **(C)** Differences in cEPCs levels of IAS according to the diabetes status (*P < 0.05 vs. diabetics).

The proportions of CD34+ cells were 0.293 ± 0.182% in HC, 0.364 ± 0.201% in CP, and 0.553 ± 0.311% in IAS patients (all P < 0.05). The proportions of CD34+/CD133+ cells were 0.098 ± 0.032% in HC, 0.113 ± 0.064% in CP, and 0.246 ± 0.142% in IAS patients (P < 0.001 for IAS vs. CP and HC; P = 0.382 for CP vs. HC) (Table 1). The proportions of CD34+/CD133+/KDR + cEPCs among total PBMCs were 0.021 ± 0.011% in HC, 0.026 ± 0.012% in CP, and 0.059 ± 0.031% in IAS patients (Figure 1A). The number of CD34+/CD133+/KDR + cEPCs was significantly higher in patients with IAS than in CP and HC (all *P* < 0.001), but no significant difference was observed in cEPCs between CP and HC (*P* = 0.401).

Multiple logistic regression showed that the numbers of cEPCs (OR 3.31, 95%CI 1.26-8.87, *p* = 0.025; IAS *vs.* CP) were an independent IAS marker after adjustment for hypertension, diabetes, smoking and CRP levels (Table 2).

**Table 2:** **Multivariate model of characteristics hypothesized to be associated with IAS (IAS *vs. *** **CP)**

**Characteristic**	**Odds ratio**	**95% CI**	***P***
Hypertension (yes *vs.* no)	1.38	0.97-2.59	0.045
Diabetes (yes *vs.* no)	1.73	1.15-2.89	0.017
Tobacco use (yes *vs.* no)	2.23	1.02-5.15	0.038
CRP (per 1 mg/l increase)	2.25	1.22-3.96	0.029
cEPCs counts (per 1% increase)	3.31	1.26-8.87	0.025

In HC, numbers of cEPCs were not different between subjects <60 (n = 39) and ≥60 years old (0.018 ± 0.009% *vs.* 0.022 ± 0.011%, *p* = 0.476). However, cEPCs numbers in IAS patients <60 (n = 60) was significantly higher than in patients ≥60 years old (0.077 ± 0.035% *vs.* 0.045 ± 0.016% *P* = 0.019) (Figure 1B). Among subjects ≥60 years old, cEPCs numbers in the IAS group were 2.1 times higher than in HC. Among subjects <60 years old, cEPCs numbers in the IAS group were 4.3 times higher than in HC. There was no difference between male and female patients (0.063 ± 0.039 vs. 0.055 ± 0.032%, *P* = 0.198). Diabetic patients had significantly less EPCs than non-diabetics (0.043 ± 0.019 vs. 0.065 ± 0.042%, *P* = 0.024) (Figure 1C).

#### Association between cEPC numbers and clinical characteristics in IAS patients

As shown in Table 3, cEPC numbers were significantly higher in patients with ≥70% stenosis than in those with <70% stenosis (0.091 ± 0.035% *vs.* 0.052 ± 0.022%, *P* = 0.012). There was no significant difference in cEPCs between 1, 2 and >2 stenosed artery (*P* = 0.578).

**Table 3:** cEPC levels according to stenosis degree and the number of symptomatic vessels in IAS patients

	**Number of patients**	**cEPCs(%)**	***P***
Stenosis			0.012
<70%	48	0.052 ± 0.022	
≥70%	60	0.091 ± 0.035	
Number of intracranial symptomatic arteries			0.578
1	30	0.065 ± 0.024	
2	26	0.058 ± 0.033	
>2	52	0.067 ± 0.044	

#### Association between cEPC numbers and plasma fibrinogen levels

**Figure 2:**
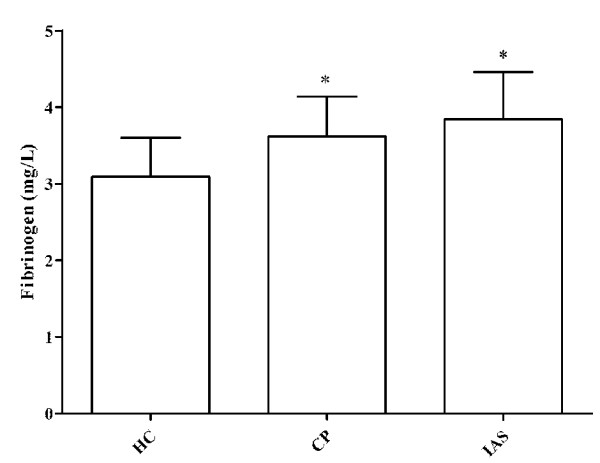
Differences in fibrinogen levels between the three groups (*P **< 0.05 *vs. *healthy controls).** Results are presented as mean ± SD.

Compared with HC, CP and IAS patients showed higher plasma fibrinogen levels (3.09 ± 0.51 mg/L vs. 3.62 ± 0.52 and 3.84 ± 0.62 mg/L, respectively) (Figure 2). No significant correlation was detected between cEPC numbers and plasma fibrinogen levels in the three groups (HC: *r =* 0.29, *P* = 0.21; CP: *r* = 0.19, *P* = 0.54; IAS: *r* = 0.23, *P* = 0.32).

## Discussion

Our study demonstrated that IAS patients had an increased mobilization of cEPCs in peripheral circulation. Moreover, cEPC levels were higher in patients with higher stenosis (≥70%). However, we did not observe any correlation between cEPC and fibrinogen levels. Nevertheless, cEPC counts could be used to assess the extent of IAS.

The association between the number of cEPCs and clinical manifestations of atherosclerosis has been previously reported. Morishita et al. [12] observed that the number of cEPCs was a marker of severity of peripheral artery diseases. In addition, Rafat et al. [21] showed that the number of EPCs was significantly higher in patients with atherosclerotic cerebral vascular disease compared with healthy controls. Chu et al. [22] showed that cEPC counts were associated with known markers of vasculopathy (HbA1c and homocysteine) in acute stroke patients. Pelliccia et al. [18] showed that cEPC numbers were associated with prognosis in patients with percutaneous coronary intervention (PCI)-treated stable angina, and that PCI-treated stable angina patients with restenosis had higher cEPC numbers [23]. On the other hand, Yoshihara et al. [30] observed no differences in circulating CD34+ cells in patients with major cerebral artery occlusion (or sever stenosis), and Sobrino et al. [31] showed that elevated cEPC numbers indicated an improved prognosis in stroke patients. This might be explained by the fact that CD34 is also present on mature endothelial cells and monocytes, and cannot be used alone to characterize bone marrow-derived immature cells. Although the exact phenotype of cEPCs is still controversial, the concomitant presence of CD34, CD133 and KDR seems to be well-supported [28,29], and we used this profile in the present study. Our results suggest that cEPCs, defined as CD34+/CD133+/KDR + cells, may indicate a disorderly growth of vascular endothelium in different intracranial lesions. Thus, these cells may be a useful marker for IAS. However, the association between cEPCs and markers of atherosclerosis remains controversial, as shown by previous studies [12,18,21-23], and the results from the present study about the lack of association between cEPC and fibrinogen levels.

However, these previous studies did not discriminate between intracranial or extracranial artery stenosis. One of our previous studies suggested that there were obvious differences between atherosclerosis lesions in intra- and extracranial cerebral large arteries [32]. Thus, ideally, intra- and extracranial atherosclerosis should be studied separately. In the present study, we observed that cEPC levels in patients with IAS were significantly higher than in HC. Moreover, cEPC numbers in IAS were significantly higher compared with stroke patients without IAS. These results indicate that abnormal EPC mobilization may play a role in the development and presence of IAS. Indeed, under normal circumstances, cEPCs participate in the formation and repair of vascular endothelial cells [7]. However, pathological conditions increasing EPCs activity might lead to excessive angiogenesis and artery narrowing. Ischemia and hypoperfusion induce endogenous VEGF production, which is an important factor for EPC mobilization [33]. However, ischemia symptoms are similar between patients with or without IAS. Therefore, we suppose that ischemia is not the only reason for higher cEPC numbers observed in patients with IAS. Our results also showed that cEPC numbers were not associated with the number of stenotic intracranial arteries, but with the degree of IAS, suggesting that stenosis degree and number of symptomatic intracranial arteries may be determined by different factors.

Increasing age has been shown to be associated with reduced cEPC levels in patients with coronary artery disease (CAD) [14]. In this present study, no difference was observed in cEPC levels between age groups (<60 and ≥60 years old) in HC. However, we observed that cEPC numbers were significantly higher in IAS patients <60 years old compared with ≥60. These results indicated that higher cEPC numbers might play a more important role in atherosclerosis progression in younger patients.

Many factors, including growth factors, proinflammatory cytokines, chemokines, hormones and lipid-lowering and antidiabetic drugs, have been regarded as important factors involved in EPC mobilization [34]. Fibrinogen is an inflammatory marker and is closely related with atherosclerosis [24-26]. To the best of our knowledge, no study focused on the association between fibrinogen levels and cEPCs mobilization. Our study showed that serum fibrinogen levels were higher in IAS patients and CP compared with HC, but that there was no difference between IAS and CP, suggesting that fibrinogen might not be a key factor stimulating cEPCs mobilization specifically in IAS. This was further supported by the lack of correlation between cEPC numbers and plasma fibrinogen levels in IAS and CP.

Studies on the relationship between cEPC numbers and coronary artery stenosis (CAS) are conflicting. It was reported that cEPC numbers were decreased in CAD with significant CAS [15]. However, Guven et al. [16] observed that cEPC numbers were higher in patients with significant CAS compared with patients without significant CAS. This might be due to the criteria used to identify specific angiogenic cell subpopulations and to evaluate CAD. These results in combination with our study suggested that the difference in the definition of cEPCs might greatly influence the correlation of cEPCs with the specific diseases such as IAS and CAS.

The present study has some limitations. First, even if we used a widely recognized method to identify cEPCs, there is a lack of standardization for this measurement, which could affect the results. Second, we cannot rule out the possibility that the elevation in CD34+ and CD133+ cells was caused by tissue ischemia inducing increased VEGF levels and peripheral blood cells mobilization. Third, it is possible that drugs taken by the patients might affect the proportions of EPCs. Fourth, we did not measure inflammation and stress markers in stroke patients, only CRP. Lastly, our sample size limits the application of our data in current clinical practice. Therefore, our results need to be confirmed in large-scale trials. Despite these limitations, our results revealed that higher cEPC numbers were closely associated with IAS.

## Conclusion

Our study demonstrated that higher cEPC numbers were involved in IAS. It also indicated that fibrinogen may not be the main mediator for cEPCs mobilization in IAS. More studies are required to assess the key factors involved in cEPCs mobilization in IAS, and to evaluate the effects of IAS treatments on cEPC numbers.

## Abbreviations

ACA: Anterior cerebral artery; BA: Basilar artery; CAD: Coronary artery disease; CAS: Coronary artery stenosis; cEPCs: Circulating endothelial progenitor cells; CI: Confidence interval; CP: Control patients; DSA: Digital subtraction angiography; EPC: Endothelial progenitor cells; HC: Healthy controls; IAS: Intracranial artery stenosis; ICA: Internal carotid artery; IL: Interleukin; MCA: Middle cerebral artery; MCP-1: Monocyte chemoattractant protein-1; OR: Odds ratio; PBMCs: Peripheral blood mononuclear cells; PCA: Posterior cerebral artery; PCI: Percutaneous coronary intervention; SD: Standard deviation; TCD: Transcranial doppler; VA: Vertebral artery.

## Competing interest

The authors declare that they have no competing interest.

## Authors’ contributions

ZL carried out the flow cytometry analysis, and drafted the manuscript. XD participated in the design of the study and performed the statistical analysis. FF and YC carried out the biochemical analysis. RW participated in flow cytometry analysis and drafting of the manuscript. YM verified and evaluated the clinical data of the patients with cerebral artery stenosis. GZ participated in the coordination of the study and drafting of the manuscript. XK conceived of the study, and participated in its design and coordination. All authors read and approved the final manuscript.

## Pre-publication history

The pre-publication history for this paper can be accessed here:

http://www.biomedcentral.com/1471-2377/13/161/prepub
